# Applying Identity Theory to Mental Health Nursing: Lessons From 1980 to 2000 and Guidance for Nurse Researchers

**DOI:** 10.1111/jpm.70144

**Published:** 2026-05-14

**Authors:** Victoria Sweetmore

**Affiliations:** ^1^ University of Derby Derby UK

**Keywords:** community mental health, deinstitutionalisation, identity theory, mental health nursing, professional identity

## Abstract

**Aim:**

To examine the potential value of identity theory as a framework for analysing professional identity in mental health nursing, using published historical and professional literature relating to the transition from psychiatric hospital to community care in England between 1980 and 2000.

**Background:**

Professional identity shapes nurses' role performance, resilience and job satisfaction. Mental health nursing identity is particularly sensitive to organisational and policy change. Identity theory offers a structured model for understanding how role identities are formed, verified and challenged in changing practice environments.

**Method:**

A theory‐informed document analysis of published historical, policy and professional literature relating to mental health nursing between 1980 and 2000 was undertaken. Key concepts from identity theory were used as an analytic lens to explore how professional identity challenges and adaptations are described in the literature.

**Discussion:**

The analysis demonstrates that identity theory provides a coherent framework for interpreting documented accounts of role disruption, identity verification and adaptive strategies during the shift from psychiatric hospital to community‐based care. The theory enables multi‐level analysis linking individual experience, organisational context and policy change.

**Conclusion:**

Document analysis indicates that identity theory has strong potential as a theoretical framework for future empirical research on mental health nursing identity, including oral history approaches.

## Accessible Summary

**What is known on the subject:**

- Professional identity is central to how mental health nurses (MHNs) see themselves and carry out their roles.
- Organisational change, such as moving from hospital‐based to community‐based care, can disrupt professional identity.
- Identity Theory offers a way to understand how people maintain or adapt their sense of self in response to change.

**What this paper adds to existing knowledge:**

- Uses the example of MHNs in England between 1980 and 2000 to show how identity theory can be applied in nursing research.
- Explains the concepts of identity verification and non‐verification and how they help us understand the effects of systemic change on nurses.
- Provides a guide for nurse researchers interested in using identity theory in their own studies.

**Implications for practice:**

- Applying identity theory can help identify situations where nurses' professional identity is challenged.
- Understanding these processes can guide the development of workplace supports that reinforce nurses' role alignment and support effective role enactment.
- The approach is relevant for all areas of nursing, not just mental health and can inform education, workforce planning and policy.

## 1 Introduction

Professional identity, that is the integration of values, beliefs, skills and self‐perceptions that define how nurses understand and enact their role, is a cornerstone of nursing practice. A well‐developed professional identity supports clinical competence, ethical decision‐making, job satisfaction and resilience, and is closely linked to patient outcomes (Johnson et al. 2012; Hunter and Cook 2018). In contrast, identity disruption, where nurses experience a mismatch between their sense of self and the expectations of their role, is associated with job dissatisfaction, burnout and workforce attrition (Trede et al. 2012; Adams et al. 2006). For these reasons, understanding the processes by which nurses construct, maintain and adapt their professional identities is essential for workforce development and the delivery of high‐quality care.

In mental health nursing (MHN), identity is shaped not only by interactions with patients and colleagues but also by wider societal attitudes toward mental illness and those who work in the field (Happell et al. 2018). This makes MHNs' professional identity especially sensitive to shifts in health policy, service delivery models and public discourse. The period from 1980 to 2000 in England represents a particularly rich case for examining these dynamics. During these two decades, mental health care underwent one of the most significant transformations in its history, with the closure of long‐stay psychiatric hospitals and the widespread implementation of community‐based care (Scull 2011; Busfield 1986). These changes, collectively known as deinstitutionalisation, fundamentally altered the institutional and organisational contexts in which MHNs practised.

The transition from large psychiatric hospital–based custodial care to multidisciplinary, community‐focused models was not merely a logistical or clinical shift; it was an identity challenge. MHNs who had worked in the structured, hierarchical environments of large institutions were suddenly required to navigate less formal, more autonomous community settings. They had to reconfigure relationships with service users, work more closely with other health and social care professionals, and adapt to new expectations of advocacy, empowerment and patient‐centred care (Cleary et al. 2012; Happell et al. 2018). Published historical and professional accounts describe varied responses to these changes, including reports of role ambiguity, loss of status and tensions between personal values and organisational priorities. These experiences were further shaped by persistent public stigma toward mental illness, stigma that sometimes extended to the professionals who provided care (Corrigan and Shapiro 2010), adding another layer of complexity to the task of maintaining a coherent and positive professional identity.

Identity theory (Stryker 1980; Burke and Stets 2009, 2023) offers a powerful conceptual framework for examining these processes. Rooted in symbolic interactionism, the theory conceptualises identity as an internalised set of role meanings, or an identity standard, that individuals strive to verify through social interaction. When feedback from others aligns with the identity standard, identity verification occurs, reinforcing the individual's self‐concept and producing positive emotions. When feedback misaligns, identity non‐verification produces emotional distress and often prompts behavioural adaptation or identity renegotiation (Burke and Stets 2009). Applied to MHN, this lens enables an analysis of how professional role identities may be challenged and renegotiated during periods of systemic change, how validation processes operate and how adaptation or resistance can be understood conceptually.

The purpose of this paper is twofold. First, it examines how identity theory can be used to analyse the development and transformation of MHNs' professional identity during the 1980–2000 period in England, a time of major structural and cultural change in mental health care. Drawing on published historical and professional literature, it demonstrates how identity theory can be used as an analytic lens to interpret documented accounts of identity challenge and adaptation. Second, it considers how the same theoretical framework can be applied more broadly across the nursing profession to understand identity formation and adaptation in diverse practice contexts. By doing so, it seeks to demonstrate that identity theory not only provides a valuable tool for historical and conceptual analysis but also offers practical insights for contemporary nursing workforce planning, education and organisational development.

Despite the centrality of professional identity to nursing practice, there remains a relative paucity of research that examines nursing identity within a robust theoretical framework, particularly from a historical perspective (Johnson et al. 2012; Hunter and Cook 2018). In the case of mental health nursing, much of the literature has focused on contemporary workforce challenges, clinical competencies, or service delivery models, with less attention given to the lived experiences of nurses during earlier periods of systemic change. Historical analyses that do exist often prioritise policy developments or institutional narratives, overlooking the personal and collective identity processes that shaped, and were shaped by, these reforms (Busfield 1986; Scull 2011). *By* applying identity theory to published historical and professional sources, this paper offers a conceptually grounded interpretive framework linking individual, organisational and policy‐level dynamics. This approach contributes a theoretically structured perspective to nursing identity scholarship, enabling a deeper understanding of how professional identities may be constructed and negotiated in changing professional landscapes. In doing so, it positions identity not only as an outcome of historical change but as a dynamic factor influencing the trajectory of nursing practice itself.

This paper forms part of a wider research programme examining mental health nursing identity across periods of systemic change. The present manuscript focuses specifically on the theoretical and conceptual foundations of that programme and does not report empirical findings. It is offered as a guide for nurse researchers who may wish to use identity theory as a conceptual framework in their own studies, whether examining historical shifts in nursing or exploring contemporary challenges in professional identity formation. By outlining the key tenets of the theory and demonstrating its application to the mental health nursing context through document‐based analysis, this paper aims to support researchers in selecting and operationalising robust theoretical models for their investigations.

## 2 Methodological Approach

This paper adopts a theory‐informed document analysis to examine the potential usefulness of identity theory as developed in the identity control model tradition associated with Burke and Stets (2009, 2023) as a framework for analysing professional identity change in mental health nursing. Document analysis is a recognised qualitative approach in which published texts are systematically examined to identify patterns of meaning, conceptual themes and interpretive insights relevant to a defined question (Bowen 2009). In this case, the question guiding the analysis was whether identity theory provides a coherent and analytically productive lens for understanding documented accounts of professional identity challenge and adaptation during major service reconfiguration.

Sources examined included published historical accounts of mental health services, nursing history texts, policy documents and peer‐reviewed professional literature addressing mental health nursing roles and practice during the period 1980–2000 in England. These materials were selected because they provide detailed descriptions of organisational change, role expectations, professional discourse and workforce experience associated with the transition from psychiatric hospital to community‐based care.

Core constructs from identity theory, including identity standards, identity verification and non‐verification, identity salience, commitment and adaptive identity processes, as defined within Burke and Stets' (2023) framework, were used as sensitising concepts to guide interpretation. The analysis involved mapping documented descriptions of role change, professional tension and adaptation strategies onto these theoretical constructs to assess conceptual fit and explanatory value. The aim was not to generate empirical findings or to test theory, but to explore its utility as an interpretive framework for nursing identity research.

## 3 Identity Theory in Brief

Identity theory is a sociological framework that explains how individuals define themselves in relation to the social roles they occupy, and how these self‐definitions influence behaviour. Originating in symbolic interactionism, it was formalised by Stryker (1980) and later developed by Burke and Stets (2009, 2023) into a model that integrates structural, cognitive and emotional dimensions of identity. Although identity theory in the Burke and Stets tradition has been widely applied in sociological and organisational research, its explicit use in nursing scholarship remains relatively limited, with most nursing identity research drawing on related but distinct theoretical traditions. For example, identity theory has been applied in nursing research to examine professional identity formation and role adaptation. Studies such as Willetts and Clarke (2014) have drawn on identity‐based frameworks to explore how nurses construct professional identity in practice contexts, while MacIntosh (2003) examined identity change in primary care nursing roles. These examples illustrate the relevance of identity‐based approaches, even where the theory is not always explicitly foregrounded.

At its core, the theory views identity as an internalised set of meanings, the identity standard, associated with a particular role (e.g., ‘I am a competent, compassionate mental health nurse’). Individuals act to verify this identity in social situations, striving for alignment between their self‐concept and feedback from others. When alignment occurs, identity verification reinforces self‐esteem, emotional stability and role commitment. When misalignment occurs, identity non‐verification produces negative emotions and may prompt behavioural adjustment or identity renegotiation (Burke and Stets 2009).

Several core concepts structure identity theory and are particularly relevant to professional roles such as mental health nursing. Role identity refers to the meanings and expectations attached to a specific social role (Stryker and Burke 2000). For a mental health nurse (MHN), this may include being a patient advocate, maintaining therapeutic relationships and demonstrating professional competence. These expectations are shaped by organisational policies, professional standards and cultural norms. Role identities are hierarchical; some are more salient than others, influencing how often they are enacted and how strongly they guide behaviour (Serpe and Stryker 2011).

Identity salience refers to the likelihood that a given identity will be invoked across situations (Stryker 1980). Commitment reflects the degree to which an identity is tied to relationships and resources; high commitment increases resistance to changing that identity (Stryker and Burke 2000). For example, a MHN deeply embedded in professional networks and community mental health advocacy may prioritise their nursing identity over other social roles.

The identity control model, first articulated by Burke (1991), conceptualises identity processes as a self‐regulating feedback loop in which individuals compare perceived feedback about their behaviour with their internal identity standard. Within this model, identity standards represent internalised role meanings and expectations, perceived self refers to one's interpretation of behaviour in context and social feedback consists of reflected appraisals from others. A comparator process evaluates whether feedback matches the identity standard, producing positive emotional responses when verification occurs and negative emotions when it does not. Where misalignment persists, behavioural adjustment or modification of identity standards may occur to restore alignment. This process operates continuously, guiding everyday role enactment and adaptation.

People hold multiple identities (e.g., nurse, parent, colleague), which can complement or conflict with each other (Burke and Stets 2009). Role conflict arises when fulfilling one identity compromises another. For MHNs, organisational pressures for efficiency may conflict with personal and professional commitments to patient‐centred care. While identity theory focuses on role‐based self‐concepts, Social Identity Theory (Tajfel and Turner 1979) addresses group‐based self‐definitions. Burke and Stets (2009) integrate both perspectives, recognising that professional identity is shaped by individual role meanings and collective group membership. This integration is particularly relevant for nursing, where belonging to the professional community provides both social support and normative guidance.

In nursing, identity theory helps explain how professional identity is formed, maintained and challenged across varying contexts. For MHNs, the theory highlights how identity verification occurs through interactions with patients, colleagues and institutions; how systemic changes (e.g., deinstitutionalisation) can trigger identity non‐verification; and how nurses may respond through role redefinition, advocacy, or withdrawal. Its emphasis on the interplay between internalised role standards and external feedback makes it a useful tool for analysing workforce resilience, retention and role satisfaction (Johnson et al. 2012; Adams et al. 2006).

## 4 Applying Identity Theory to Mental Health Nursing, 1980–2000

This piece draws on historical sources, published literature and theoretical reasoning to illustrate how identity theory can be applied to this period of significant systemic change in mental health care. The period from 1980 to 2000 in England marked one of the most significant shifts in the history of mental health care. This was a time when large psychiatric hospitals, many of which had been operating for over a century, were closed as part of the national move toward community‐based care (Scull 2011; Busfield 1986). For MHNs, this change was not simply a matter of adapting to new workplaces; it is frequently characterised in historical and professional literature as involving substantial role disruption and professional uncertainty (Busfield 1986; Scull 2011; Hannigan and Allen 2011; Traynor 2017).

In institutional settings, MHNs' role identities were clearly structured within a stable organisational hierarchy. There were established routines, defined responsibilities and a professional culture shaped by the traditions of asylum nursing (Nolan 1993). Deinstitutionalisation disrupted these structures, requiring MHNs to work in community teams, primary care partnerships and patient homes. This shift necessitated the adoption of new role meanings: moving from a custodial, institutionally defined caregiver identity toward one centred on autonomy, multidisciplinary collaboration and patient empowerment (Happell et al. 2018; O'Brien 2001). The expansion of the Community Psychiatric Nurse (CPN) role was one of the clearest expressions of this shift in practice context and role identity. Historical and professional sources describe how community psychiatric nursing moved from a marginal and experimental function in earlier decades to a central component of service delivery by the 1980s and 1990s, with nurses undertaking follow‐up work, case management and therapeutic interventions outside hospital settings (Hunter 1974; Busfield 1986; Burns 2004; Gournay et al. 1998). This role repositioned nurses across organisational and professional boundaries, often with increased autonomy but also with uneven preparation and continuing role ambiguity (Nolan 1993; Davis and Keen 1999).

From an identity theory perspective, the CPN role can be understood as a site of intensified identity negotiation, where established institutional role meanings were reworked in more dispersed, multidisciplinary and community‐facing environments. In identity theory terms, this period can be understood as involving significant role redefinition. The identity standard for a MHN, the internalised expectations of what it meant to be ‘a good mental health nurse’, may become no longer fully aligned with the external conditions of practice. Published professional and historical accounts describe circumstances consistent with identity non‐verification (Burke and Stets 2009), where social feedback from colleagues, managers and patients did not consistently reinforce previously established professional role meanings.

### 4.1 The Emotional Impact of Identity Non‐Verification

Burke and Stets' (2009, 2023) model emphasises that misalignment between the identity standard and social feedback produces emotional responses. Historical accounts and professional commentary from the period describe reports of MHNs experiencing frustration, loss, or even grief for the closure of long‐standing institutions and the erosion of certain aspects of professional camaraderie (Traynor 2017). Other sources note feelings of anxiety in navigating new professional boundaries in the community, where visibility, accountability and patient relationships were reconfigured (Roche et al. 2014).

In some accounts, identity disruption is described as having energising effects. Nurses who embraced the principles of patient‐centred care are described as using the disruption as an opportunity to strengthen their professional commitment, redefining their identity standard to prioritise advocacy, flexibility and holistic care (Cleary et al. 2012; Happell and Cutcliffe 2011). In other accounts, however, a persistent gap between self‐concept and the realities of community work is associated with disengagement or attrition (Traynor 2017; Roche et al. 2014), a process predicted by identity theory when non‐verification is repeated without resolution (Burke 1991).

### 4.2 Identity Salience and Commitment Under Pressure

During this transition from psychiatric hospital–based to community‐focussed ways of working, the salience of the MHN identity can be understood as being tested. For those with high commitment, whose professional role was deeply tied to valued relationships, community networks or a strong sense of vocation, identity theory would predict that identity disruption is more likely to prompt active adaptation rather than withdrawal, as suggested by the work of Stryker and Burke (2000). Published accounts of community service development describe adaptation strategies consistent with this pattern, often involving alternative sources of affirmation, such as patient appreciation or peer support within newly formed community mental health teams (Hannigan and Allen 2011; Happell et al. 2018; Cleary et al. 2012). Conversely, for nurses with lower commitment or whose networks were weakened by institutional closure, the theory suggests that decline in salience may *be associated* with professional identity erosion. This aligns with Serpe and Stryker's (2011) finding that when the costs of maintaining an identity outweigh its rewards, individuals may de‐prioritise that identity.

### 4.3 Meso‐Level Dynamics: Teams, Organisations and Policy

At the meso level, organisational culture is widely described as playing a pivotal role in shaping identity verification opportunities. Community mental health teams are described in the literature as varying widely in their capacity to support MHNs through this transition. Some are characterised as fostering inclusive, collaborative environments where nurses' expertise was recognised alongside that of psychiatrists, social workers and occupational therapists, providing consistent positive feedback and identity reinforcement (Hannigan and Allen 2011). Others are described as being dominated by medical or managerial priorities that undervalued nursing contributions, creating recurring non‐verification and emotional strain (Hannigan and Allen 2011; Traynor 2017).

Policy‐level changes also are described as acting as macro‐to‐meso filters. Government directives promoting efficiency and cost‐containment sometimes clashed with nurses' personal and professional identity rooted in relational care (Department of Health 1990). Identity theory helps explain the resultant tensions: published accounts describe circumstances consistent with situations in which nurses received mixed social feedback, some of which supported their identity standards (e.g., praise from patients) and some of which undermined them (e.g., managerial criticism for spending ‘too much time’ with service users).

### 4.4 Strategies of Adaptation and Resistance

Historical commentary and nursing literature describe a range of strategies through which mental health nurses navigated identity challenges during this period. These strategies can be interpreted through identity theory as different mechanisms for restoring alignment between identity standards and social feedback. One commonly described pattern is behavioural adjustment, in which work practices are modified to align with emerging community care models, for example through increased use of home visits or the development of case management skills. A second pattern is identity standard recalibration, where the meaning of being a ‘good mental health nurse’ is redefined to incorporate new responsibilities and values, such as advocacy and interprofessional leadership. A third pattern is identity protection, in which practitioners seek subcontexts where identity verification is more likely, for example by prioritising relationships with supportive colleagues or focusing on patient interactions that affirm core professional values. These interpretive patterns correspond closely to Burke's (1991) prediction that when non‐verification persists, individuals will adjust behaviour, revise identity standards, or seek alternative sources of validation.

### 4.5 Long‐Term Implications

The identity shifts described in published accounts of MHNs between 1980 and 2000 have enduring relevance. They highlight the importance of designing organisational and policy environments that enable identity verification, through recognising nurses' contributions, supporting interprofessional collaboration and aligning performance measures with care values. Identity theory suggests that when verification opportunities are embedded in the work environment, professional commitment is strengthened, resilience is enhanced and attrition is reduced (Burke and Stets 2023). By situating historical accounts of MHNs' experiences within this theoretical framework, it is possible to see that identity was not a passive casualty of deinstitutionalisation but an active, negotiated resource. Published literature from the period describes nurses as engaging in ongoing identity work, balancing personal values, professional standards and organisational realities.

## 5 Implications for Contemporary Mental Health Nursing

The professional pressures experienced by MHNs during the 1980–2000 period such as organisational change, evolving role expectations and competing policy priorities have clear parallels in contemporary practice. Today's mental health services operate within a landscape shaped by digital health innovations, persistent resource constraints, heightened public health demands and an increased emphasis on integrated, community‐based models of care (Bee et al. 2015; Ross et al. 2020). Such developments require nurses to adapt their role identities, navigate new interprofessional dynamics and reconcile emerging clinical tools with existing values and practices.

Identity theory offers a structured framework for examining these processes in several complementary ways. First, it supports analysis of how policy change affects identity alignment. New health policies alter the social expectations attached to nursing roles, and the identity control model provides a way for researchers and practitioners to examine how such shifts influence alignment between role identities, personal values and professional group membership (Burke and Stets 2009; Hunter and Cook 2018). Second, the framework helps identify sources of identity non‐verification. Persistent mismatches between a nurse's identity standard and the feedback they receive, for example, managerial criticism or conflicting performance metrics, are associated in the literature with emotional distress, burnout and attrition (Johnson et al. 2012; Adams et al. 2006). Mapping these sources of non‐verification can inform organisational and policy‐level responses. Third, identity theory provides a conceptual basis for designing organisational supports that strengthen identity verification. Structures such as reflective practice groups, mentorship schemes and recognition systems can reinforce positive social feedback and validate professional role identities (Maben et al. 2012). Framing these interventions in identity terms ensures they address not only skill development but also identity affirmation.

Contemporary debates about the regulation and scope of mental health nursing practice provide a further example of identity pressure. Recent professional commentary has highlighted concerns about increasing genericisation within nursing standards and education, with greater emphasis on cross‐field and physical health competencies and less explicit articulation of mental health nursing distinctiveness. Contemporary service environments are also increasingly shaped by managerial performance cultures, administrative intensification and target‐driven models of care, which published analyses suggest can constrain relational practice and professional autonomy in mental health nursing (Ramon 2008; Jones 2020; Health Foundation 2020). From an identity theory perspective, such environments may increase the frequency of identity non‐verification experiences where organisational feedback systems reward throughput and metric compliance more strongly than relational and therapeutic role meanings.

At the same time, role expansion through advanced practice, non‐medical prescribing and nurse practitioner pathways is reshaping role boundaries and expectations. Contemporary scholarship also critiques resilience and burnout discourses for shifting responsibility for systemic pressures onto individual practitioners (Maiese 2022; Neilson 2020), a pattern that identity theory would interpret as increasing pressure on individuals to preserve identity standards under structurally non‐verifying conditions. Emerging digital and AI‐supported service models are also beginning to reshape expectations of nursing work (Department of Health and Social Care 2025; Shaw and Glover 2024), potentially introducing new identity standards around technological competence alongside traditional relational roles. From an identity theory perspective, these developments can be understood as creating new identity verification demands, as role meanings, standards and sources of professional validation are renegotiated. In this way, the theory moves beyond abstract analysis, offering a practical diagnostic and developmental tool for contemporary mental health nursing.

## 6 Relevance to Nursing More Broadly

Although this paper focuses on the mental health nursing context, beyond the mental health nursing context discussed above, identity theory has applicability across nursing specialisms. Across specialist fields, nurses frequently develop sub‐identities alongside their overarching professional identity. Nurses working in areas such as intensive care, palliative care, or perioperative nursing often develop strong specialist sub‐identities that can be highly salient, shaping clinical decision‐making, team roles and professional development trajectories (MacIntosh 2003). Identity theory provides a framework for examining how such sub‐identities are formed, maintained and integrated within the wider nursing role.

The framework is also relevant to how nurses negotiate organisational change. Service reconfigurations, mergers and funding restructures can prompt identity renegotiation across nursing fields. Understanding these processes through the lens of identity verification and non‐verification can help leaders and researchers interpret role‐related stress and design smoother transitions (Traynor 2017; Burke and Stets 2023). Interprofessional dynamics represent a further area of application. Nurses' sense of value and belonging is shaped by their position within multidisciplinary teams. Identity theory supports analysis of how interprofessional hierarchies, collaboration patterns and mutual recognition influence professional self‐concept and job satisfaction (Reeves et al. 2010).

Embedding identity theory in nursing research and education could therefore enhance understanding of role adaptation, resilience and workforce sustainability across diverse practice areas. It can inform curricula that prepare nurses for the identity work involved in career progression, specialisation and interprofessional collaboration, ensuring they have the conceptual tools to navigate change without eroding their sense of professional self.

## 7 Limitations

This paper is intended as a theoretical and illustrative exploration of how identity theory can be applied to mental health nursing, rather than as a presentation of empirical findings. While examples from the 1980–2000 period have been used to demonstrate the theory's application, these are drawn from published historical accounts and professional literature. As such, the discussion is necessarily selective, highlighting aspects of the historical context that align clearly with identity theory's core concepts. This may not fully capture the breadth and diversity of MHNs' experiences during the period. Furthermore, the illustrative focus on England's mental health system means that contextual factors specific to other countries and healthcare systems are not addressed here; adaptation would be required before applying the framework internationally.

Finally, while identity theory offers a robust and versatile analytical lens, it is only one of several sociological and psychological approaches that could illuminate the dynamics of nursing identity. The aim here is not to propose it as the sole or definitive framework, but to demonstrate its potential value for nurse researchers and to encourage critical consideration alongside alternative models.

## 8 Implications for Research

The conceptual application outlined in this paper indicates the potential value of identity theory as a framework for examining nursing identity, both historically and in contemporary contexts. For nurse researchers, adopting this lens can facilitate nuanced analysis of how role meanings, personal values and professional group memberships interact under conditions of organisational or societal change. Future studies could build on this approach by applying the identity control model to diverse nursing populations, exploring how identity verification and non‐verification processes operate across specialisms, career stages and healthcare settings. Longitudinal research could be particularly valuable for capturing identity trajectories over time, especially during periods of systemic reform or professional transition. By combining historical narrative with robust theoretical tools, such research can enrich nursing scholarship, inform workforce development and support the creation of identity‐affirming practice environments.

## 9 Conclusion

Identity theory provides a robust, multi‐level framework for understanding how nurses construct, maintain and adapt their professional identity within shifting social, organisational and historical contexts. Analysis of published historical and professional literature relating to MHNs during 1980–2000 illustrates how role disruption, societal attitudes and organisational structures can be interpreted as shaping identity processes within this framework. Documented accounts from this period are consistent with identity theory constructs of verification, non‐verification and adaptive identity work.

Applying this theoretical lens across the nursing profession highlights its potential for both research and practice. By diagnosing where and how identity verification is disrupted, organisations may be better positioned to implement supportive interventions that strengthen professional commitment and morale. In turn, this has the potential to enhance retention, improve job satisfaction and foster high‐quality patient care. Ultimately, identity theory offers a way to bridge the gap between abstract sociological concepts and the practical realities of nursing, helping to create identity‐affirming work environments that benefit both nurses and the people they care for. On this basis, document analysis supports the use of identity theory as a theoretical framework for the next stage of research, including planned oral history investigation of mental health nursing identity.

## Funding

The author has nothing to report.

## Conflicts of Interest

The author declares no conflicts of interest.

## Data Availability

Data sharing not applicable to this article as no datasets were generated or analysed during the current study.
